# A Novel DLLME-Based Approach for the Spectrophotometric Determination of Mercury in Environmental Samples Using the Fe(II) Phthalocyanine Sensor

**DOI:** 10.3390/molecules30214192

**Published:** 2025-10-27

**Authors:** Yasemin Çağlar

**Affiliations:** Department of Genetic and Bioengineering, Giresun University, 28200 Giresun, Türkiye; yasemin.caglar@giresun.edu.tr; Tel.: +90-454-310-40-16

**Keywords:** Hg^2+^, DLLME, phthalocyanine, Uv-Vis spectroscopy

## Abstract

In the present investigation, a novel dispersive liquid–liquid microextraction (DLLME) method was developed for the spectrophotometric determination of Hg^2+^. Fe(II) phthalocyanine (Fe(II)Pc) was employed as the sensor, chloroform (300 µL) as the extraction solvent, and ethanol (700 µL) as the dispersive solvent. Following the formation of the Hg^2+^:Fe(II)Pc complex, the sample was centrifuged at 1000 rpm for 2 min. The aqueous phase was discarded, and the extraction phase was diluted to 250 µL with methanol and transferred into a 250 µL quartz cell for spectrophotometric measurement at 276 nm. The method exhibited a linear range of 1–20 µg/L, with limits of detection (LOD) and quantification (LOQ) calculated as 1.44 µg/L and 4.80 µg/L, respectively. The enrichment factor was determined to be 105, and the optimum pH for the procedure was 2.0.

## 1. Introduction

Mercury (Hg) is widely acknowledged as one of the most hazardous heavy metals and has been the focus of extensive research and regulatory attention worldwide because of its high toxicity and potential to cause severe ecological disruption. The adverse impacts of mercury on biological systems are governed by several determinants, such as its chemical speciation, level of exposure, route of entry, and host susceptibility [1,2]. In humans, major exposure routes comprise dietary consumption, occupational settings, domestic applications, and products containing mercury [3,4,5]. Mercury occurs in the environment in three major forms: elemental mercury (Hg^0^), inorganic species (Hg_2_^2+^/Hg^2+^), and methylmercury (HgCH_3_^+^). Although its environmental concentrations are generally low, mercury has a strong potential for bioaccumulation through the food chain and, consequently, poses significant risks to human health and ecosystems, particularly in aquatic environments [6,7,8,9]. Populations with traditionally high seafood consumption are therefore more susceptible to one of the most critical routes of exposure [10,11,12]. Mercury exposure affects the renal, immune, and nervous systems, and at high concentrations, it may cause severe physiological damage [13,14,15,16,17,18]. Acute intoxication can provoke gastrointestinal disturbances and may even be life-threatening [19].

Although the severe toxicity of mercury is well recognized, mercury and its derivatives are still extensively utilized in various industrial processes and consumer products [20,21,22]. Reliable quantification of trace metals such as mercury in complex environmental matrices often necessitates an effective sample pre-treatment step [23,24,25,26]. Liquid-phase microextraction techniques such as immersed single-drop microextraction (I-SDME) [27], headspace single-drop microextraction (HS-SDME) [28], and hollow fiber-based liquid–liquid-liquid microextraction (HF-LLLME) [29] are commonly employed for this purpose. Among these methods, dispersive liquid–liquid microextraction (DLLME) has emerged as one of the most efficient sample preparation techniques for trace analysis of organic and inorganic pollutants in environmental and biological matrices [30,31,32,33,34]. Since its introduction in 2006, DLLME has gained widespread attention due to its simplicity, rapidity, low solvent consumption, and high preconcentration factor compared with conventional extraction methods [35]. In DLLME, a small volume of extraction solvent is rapidly dispersed into an aqueous sample with the aid of a disperser solvent, forming a fine cloudy solution that facilitates rapid mass transfer of the target analytes into the extraction phase. After phase separation, typically by centrifugation, the enriched analytes can be determined using various analytical instruments such as UV–Vis spectrophotometry, FAAS, ICP-MS, or HPLC [36,37,38,39,40,41]. DLLME is now considered a powerful and versatile technique for the determination of trace levels of heavy metals, including mercury, in complex water samples [25,42,43,44,45,46,47].

Spectroscopic methods have been validated and successfully applied for the analysis of mercury in real samples. Spectrophotometry [48], cold vapor atomic fluorescence spectroscopy (CV-AFS) [49], cold vapor atomic absorption spectrometry (CV-AAS) [50], inductively coupled plasma mass spectrometry (ICP-MS) [51], inductively coupled plasma optical emission spectrometry (ICP-OES) [52], and spectrofluorimetry [53,54,55,56] are among the techniques reported in the literature. Owing to its simplicity, low cost, ease of application, lack of need for specialized expertise, and time efficiency, UV–Vis spectroscopy can be readily integrated with the DLLME method. In this context, employing UV–Vis spectroscopy for the selective and sensitive determination of Hg^2+^ represents an effective approach [57,58,59,60].

In this study, an original dispersive liquid–liquid microextraction (DLLME) method was developed and validated for the spectrophotometric determination of Hg^2+^ in water samples, employing the Fe(II) phthalocyanine (Fe(II)Pc) as a sensor. In the DLLME procedure, the choice of chelating reagent is a critical parameter influencing extraction efficiency. Phthalocyanines are highly suitable compounds for the development of metal sensors [61,62]. The four peripheral side chains containing nitrile groups (soft bases) are expected to enhance the selective binding affinity toward mercury (Hg^2+^), which is classified as a soft acid. The central Fe(II) stabilizes the phthalocyanine by preserving the rigidity of the macrocycle, improving resistance to environmental conditions, and ensuring consistent spectroscopic responses, thereby making Fe(II)Pc a reliable sensor for Hg^2+^ detection.

## 2. Results and Discussion

### 2.1. Optimization of DLLME

#### 2.1.1. Kind and Volume of Extractive/Disperser Solvents

**Figure 1:**
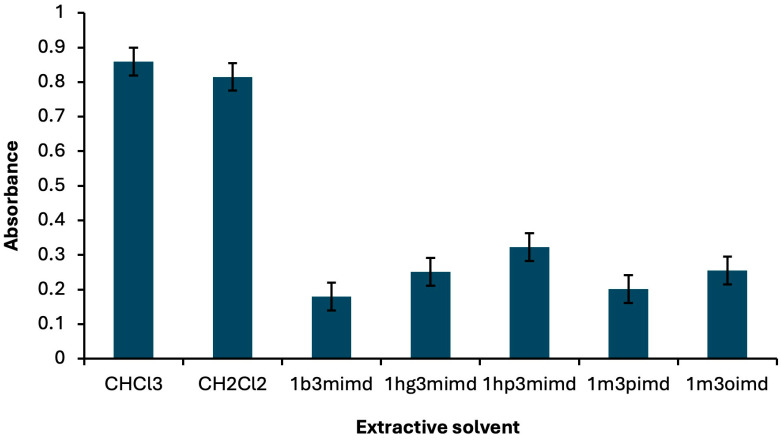
Absorbance changes observed with different extractive solvents. Extraction conditions: sample volume, 5.00 mL; disperser solvent, 700 μL ethanol; 250 µL of Fe(II)Pc (5.00 × 10^−6^ M); pH, 2.0; centrifugation conditions: 1000 rpm, 2 min. Error bars show the variability (±SD) for three replicate determinations (*n* = 3).

In the DLLME procedure, the selection of the extractive solvent is of critical importance, as the extraction efficiency strongly depends on this factor. The extractive solvent should possess a high extraction capacity for the analyte, exhibit very low solubility in water, and have a density greater than that of water [63,64]. In this study, to optimize the extractive solvent kind, chloroform, dichloromethane, 1-butyl-3-methylimidazolium hexafluorophosphate, 1-hexyl-3-methylimidazolium hexafluorophosphate, 1-heptyl-3-methylimidazolium hexafluorophosphate, 1-methyl-3-octylimidazolium hexafluorophosphate, and 1-butyl-3-pentylimidazolium hexafluorophosphate were tested. The absorbance reached its maximum value when chloroform was employed as the extractive solvent (Figure 1).

In DLLME, determining the appropriate volume of extractive solvent is an important parameter, as it provides maximum extraction efficiency with minimal solvent consumption [65]. To determine the optimal extractive solvent volume for the DLLME method, enrichment factors obtained using different volumes of chloroform were compared. The optimal extractive solvent volume for this method was found to be 300 µL.

**Figure 2:**
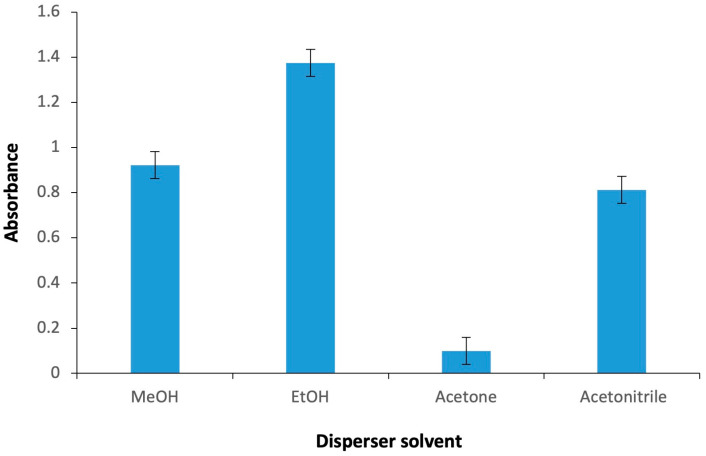
Absorbance changes observed with different extractive solvents. Extraction conditions: sample volume, 5.00 mL; extractive solvent, 300 μL chloroform; 250 µL of Fe(II)Pc (5.00 × 10^−6^ M); pH, 2.0; centrifugation conditions: 1000 rpm, 2 min. Error bars show the variability (±SD) for three replicate determinations (*n* = 3).

The choice of disperser solvent significantly affects both the stability of the cloudy phase and the enrichment factor achieved. The disperser solvent not only facilitates the rapid dispersion of the extraction solvent into the aqueous phase but also acts as a medium that bridges the polarity differences between the aqueous solution and the extraction solvent. This bridging role is essential for the efficient transfer of analytes into the extraction phase and for maintaining the reproducibility and effectiveness of the overall microextraction process [66,67]. To determine the most appropriate disperser solvent for the preconcentration of Hg^2+^, four solvents (acetone, acetonitrile, ethanol, and methanol), all of which are miscible with both aqueous and organic phases, were evaluated in this procedure. The experimental results demonstrated that a sedimented phase could be obtained with all four disperser solvents; however, the signal responses varied (Figure 2). The most notable finding was that the highest signal response was obtained with ethanol, which was therefore selected as the disperser solvent for the proposed DLLME procedure.

The disperser solvent volume plays a fundamental role in the formation of the cloudy phase within the microextraction tube, which represents a ternary system. When the volume of the disperser solvent is too low, the cloudy solution does not form efficiently, resulting in reduced extraction efficiency. Conversely, if the disperser solvent volume is excessively high, the solubility of the complex increases, which again diminishes the extraction efficiency [68]. To optimize the disperser solvent volume, enrichment factors were compared using different volumes of ethanol as disperser solvents. The optimal volume for the proposed method was established as 700 µL.

#### 2.1.2. Effect of pH

**Figure 3:**
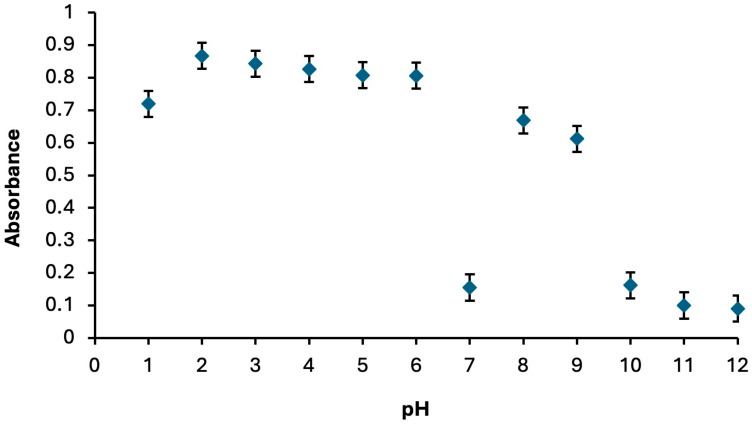
Absorbance changes observed with different extractive solvents. Extraction conditions: sample volume, 5.00 mL; extractive solvent, 300 μL chloroform; disperser solvent, 700 μL ethanol; 250 µL of Fe(II)Pc (5.00 × 10^−6^ M); centrifugation conditions: 1000 rpm, 2 min. Error bars show the variability (±SD) for three replicate determinations (*n* = 3).

pH is a key parameter influencing both metal–sensor complex formation and the extraction process [69]. In the spectrophotometric determination of Hg^2+^, the optimum pH was established by preparing a series of Hg^2+^:Fe(II)Pc complexes at pH values ranging from 1.0 to 12.0. The maximum absorbance intensity was observed at pH 2 for the Fe(II)Pc sensor (Figure 3).

#### 2.1.3. Role of Centrifuge Parameters

**Figure 4:**
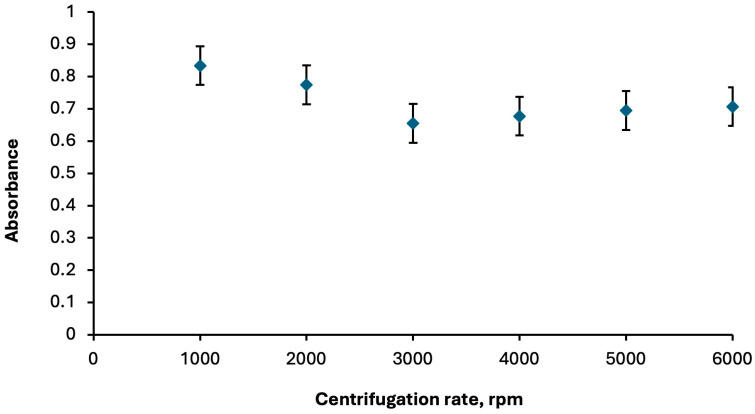
Optimization of centrifugation rate for phase separation. Extraction conditions: sample volume, 5.00 mL; extractive solvent, 300 μL chloroform; disperser solvent, 700 μL ethanol; 250 µL of Fe(II)Pc (5.00 × 10^−6^ M); pH, 2.0; centrifugation conditions: 2 min. Error bars show the variability (±SD) for three replicate determinations (*n* = 3).

**Figure 5:**
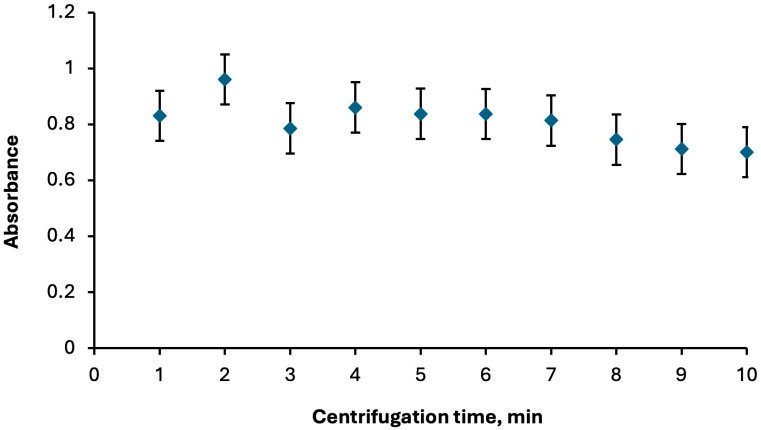
Optimization of centrifugation time for phase separation. Extraction conditions: sample volume, 5.00 mL; extractive solvent, 300 μL chloroform; disperser solvent, 700 μL ethanol; 250 µL of Fe(II)Pc (5.00 × 10^−6^ M); pH, 2.0; centrifugation conditions: 1000 rpm. Error bars show the variability (±SD) for three replicate determinations (*n* = 3).

The centrifugation step in DLLME enables efficient phase separation of the cloudy solution formed during extraction. Appropriate centrifugation speed and time allow the extraction phase to settle completely, thereby enhancing enrichment, improving reproducibility, and ensuring that the analyte-rich phase can be readily collected for subsequent spectroscopic analysis. The centrifugation step was optimized by evaluating speeds between 1000 and 6000 rpm and times of 1 to 10 min. The best performance was achieved at 1000 rpm for 2 min (Figure 4 and Figure 5, respectively).

#### 2.1.4. Compex Stability

**Figure 6:**
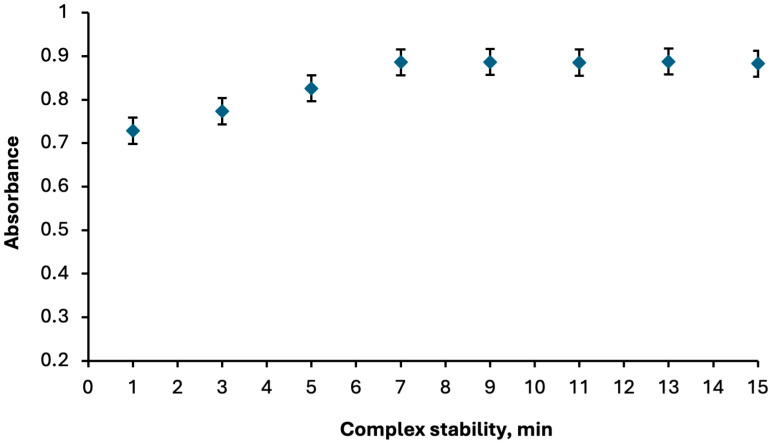
Time-dependent absorbance variation in the Hg^2+^:Fe(II)Pc (1:1) complex.

The absorbance variation in the Hg^2+^:Fe(II)Pc complex at 276 nm was monitored within the time interval of 1–15 min in order to evaluate its stability. The results revealed that the complex gradually increased in stability and reached a steady state after the seventh minute. Once equilibrium was established, the Hg^2+^:Fe(II)Pc complex remained stable for approximately 8 min. without exhibiting significant fluctuations in absorbance (Figure 6). This observation indicates that the formed complex possesses sufficient temporal stability under the applied experimental conditions, which is a critical factor for ensuring reliable spectrophotometric measurements in analytical applications.

#### 2.1.5. Effect of Fe(II)Pc Sensor Concentration

The effect of sensor concentration on the absorbance was investigated by varying the Fe(II)Pc concentration in the range of 1.00 × 10^−6^ to 1.00 × 10^−4^ M, while keeping all other experimental parameters constant. The absorbance reached its highest value at 5.00 × 10^−6^ M, which was selected as the optimum sensor concentration for subsequent measurements.

### 2.2. Interferences

In order to assess the applicability of the proposed method to real samples, the potential interfering effects of various cations and anions were systematically examined, and their tolerance limits were established. For this purpose, solutions containing 5.00 × 10^−6^ M sensors and an equimolar amount of Hg^2+^ were spiked with different concentrations of foreign ions to prepare a series of mixtures. These mixtures were subjected to the proposed microextraction procedure, and the absorbance intensities measured at 276 nm were compared with those of a reference solution containing only the sensor and Hg^2+^. The tolerance limit was defined as the maximum concentration of a foreign ion that produced a deviation of less than 5% from the reference absorbance. The obtained values are summarized in Table 1.

### 2.3. Analytical Figures of Merit

The linear dynamic range of the proposed methods for the spectrophotometric determination of mercury was determined using spectrophotometric titration (Supplementary Figure S1) and was found to be 1.00–20.00 µg/L with a correlation coefficient (R^2^) of 0.9978 (Supplementary Figure S2). The limit of detection (LOD) and limit of quantification (LOQ) of the proposed DLLME method were estimated using the equations LOD = 3σ/S and LOQ = 10σ/S, where σ represents the standard deviation of the response and S is the slope of the calibration curve. In this study, σ was calculated from the standard deviation of absorbance values obtained from 11 blank samples. Based on these calculations, the LOD and LOQ were determined to be 1.44 µg/L and 4.80 µg/L, respectively. The enrichment factor, defined as the ratio of the slopes of the calibration curves for Hg^2+^ obtained before and after the extraction procedure, was calculated to be 105. The precision of the developed method was assessed under both intra-day and inter-day conditions by analyzing Hg^2+^ at a concentration level of 10 µg/L. The relative standard deviations (RSDs) were observed in the range of 1.27–2.68% for intra-day analyses and 1.09–3.01% for inter-day analyses (*n*:6) (Table 2).

Since the Hg^2+^ concentrations in the environmental water samples were below the detection limit of the proposed DLLME method, the analysis was carried out through spiking experiments based on the recovery of added Hg^2+^ ions into the sample matrices. For this purpose, different amounts of analyte ions were added to 25 mL portions of three different water samples, and the proposed microextraction procedure was applied using 5.00 mL of each sample. The results are presented in Table 3, showing recovery values ranging from 94.00% to 99.00%.

### 2.4. Comparison with Other Studies

Table 4 outlines selected reports addressing the determination of Hg^2+^ through the DLLME technique. Nemati et al. (2023) [5] developed a DES based DLLME method for Hg^2+^ extraction using 4,4′-bis(dimethylamino)thiobenzophenone (TMK) as a ligand and a coumarin–octanoic acid solvent system. TMK is a commonly used ligand for mercury determination. In contrast, the proposed method employs Fe(II)Pc for the first time in mercury determination. Although the TMK based method achieved good extraction efficiency, with an enrichment factor of 200, its performance strongly depends on the solvent composition, as conventional DES systems fail to stabilize the Hg–TMK complex. This solvent-specific limitation reduces the method’s applicability and increases its procedural complexity. Moreover, due to the negative influence of halide ions on the stability of the Hg^2+^–TMK complex, the addition of sodium sulfate (5.0% *w*/*v*) was required in their procedure. In contrast, the Fe(II)Pc based system developed in this study provides high extraction efficiency without the need for any additional chemical agents such as salts. In the study by Gharehbaghi et al. (2009) [70], an ionic liquid based DLLME method was developed for the spectrophotometric determination of Hg^2+^ using 4,4′-bis(dimethylamino)thiobenzophenone (TMK) as a chelating ligand and (1-hexyl-3-methylimmidazolium bis(trifluormethylsulfonyl)imid) [Hmim][Tf_2_N] as the extraction solvent. Although the method demonstrated acceptable analytical performance, the use of TMK, a widely employed ligand for mercury determination, reduces the originality of the approach. Furthermore, the addition of sodium nitrate (NaNO_3_) to improve extraction efficiency and sodium dodecyl sulfate (SDS) to prevent the adhesion of ionic liquid droplets to the test tube walls after centrifugation introduced additional preparation steps, thereby increasing the cost, time requirements, and environmental impact of the procedure. Additionally, the centrifugation time reported in their study was 4 min, whereas the proposed Fe(II)Pc based DLLME method achieves efficient phase separation within only 2 min, thereby enhancing practical applicability. In the study by Ripoll et al. (2023) [57], a NADES-based DLLME method was developed for the determination of inorganic and organic mercury species (MeHg^+^, EtHg^+^, PhHg^+^, and Hg^2+^). Although the method successfully achieved mercury speciation using a hydrophobic NADES system (decanoic acid:DL-menthol, 1:2 molar ratio), it exhibits several drawbacks in terms of operational efficiency and simplicity. In addition to the standard centrifugation step, a 3 min vortexing process was required to ensure NADES dispersion, thereby increasing the total analysis time. Adjusting the sample to pH 12 may also cause hydrolysis or transformation of mercury species, affecting extraction reliability. Furthermore, the use of dithizone as a conventional ligand and the incorporation of EDTA during liquid chromatography (LC) analysis reduce the method’s novelty, cost-effectiveness, and environmental compatibility. Compared with the proposed Fe(II)Pc based DLLME method, the longer centrifugation time and multi-step procedure make this approach less efficient in terms of time, resources, and practicality. Hossein-Poor-Zaryabi et al. (2014) [71] developed a DLLME–UV–Vis method for the determination of Hg^2+^ using dithizone as a chelating reagent, ethanol as a disperser solvent, and chloroform as the extraction solvent. The optimum extraction pH reported in their study was 1.80, which is consistent with the pH 2.00 condition employed in the Fe(II)Pc based DLLME method. Both methods operate under highly acidic conditions, ensuring that Hg^2+^ ions remain in soluble form and preventing the formation of mercury hydroxide precipitates. However, the dithizone-based method achieved a relatively low enrichment factor (39) compared to 105 obtained with the Fe(II)Pc based system, indicating significantly higher extraction efficiency and analytical sensitivity in the present study. Moreover, the use of dithizone, a conventional complexing reagent widely applied in mercury determination, limits the originality of the previous approach. In contrast, Fe(II)Pc has been employed for the first time as a sensing and extraction agent for Hg^2+^, providing enhanced selectivity, methodological innovation, and improved extraction efficiency. In the DLLME method developed by Çağlar et al. (2018) [72], the quaternized Fe(II)phthalocyanine (q-Fe(II)Pc) compound, the water-soluble form of the sensor used in present study, was employed as the sensing agent, and 1-heptyl-3-methylimidazolium and acetonitrile were used as the extraction and dispersive solvents, respectively. The Fe(II)Pc used in the present study exhibits a lipophilic nature which, compared to the ionic character of q-Fe(II)Pc, enables the formation of neutral and hydrophobic Hg^2+^:Fe(II)Pc complexes that efficiently transfer into the organic phase, thereby enhancing extraction efficiency. The previously reported method exhibited a linear range of 0.05–1.00 µg/mL (R^2^ = 0.9960) with LOD and LOQ values of 0.01 and 0.03 µg/mL, respectively. In contrast, the method developed in this study operates within a linear range of 1.00–20.00 µg/L (R^2^ = 0.9978) and provides LOD and LOQ values of 1.44 and 4.80 µg/L, respectively. Overall, these results indicate that the lipophilic Fe(II)Pc compound employed in this work offers superior extraction performance and analytical sensitivity compared to its water-soluble derivative, making it more suitable for DLLME-based preconcentration of Hg^2+^ ions.

Compared with these studies, the method developed in the present work, which employs Fe(II)Pc as a novel sensor together with chloroform and ethanol as the extraction and dispersive solvents, respectively, provides a narrower linear range (1.00–20 µg/L) but demonstrates satisfactory sensitivity with a LOD of 1.44 µg/L, LOQ of 4.80 µg/L, and a notably high enrichment factor of 105. Furthermore, the use of a phthalocyanine based ligand, which remains scarcely explored in DLLME, highlights the originality of the proposed method. Although the sensitivity parameters of some previous studies are superior, the present work contributes a unique approach by demonstrating the applicability of Fe(II)Pc as a selective sensor, thereby addressing an existing gap in DLLME based mercury determination.

## 3. Materials and Methods

### 3.1. Reagents and Instrumentation

Fe(II)Pc was synthesized in accordance with a previously published procedure [72]. Analytical grade reagents and solvents were procured from Merck (Darmstadt, Germany) and Sigma-Aldrich (Taufkirchen, Germany). A stock solution of Hg^2+^ was prepared from nitrate salts in methanol followed by acidification with HNO_3_. The Fe(II)Pc sensor solution was prepared daily by dissolving a precisely weighed amount of the compound in methanol. The pH required for extraction was controlled and adjusted with buffer systems composed of citric acid, NaOH, and HCl.

All details for instrumentation were given in Supplementary File.

### 3.2. DLLME Procedure

**Figure 7:**
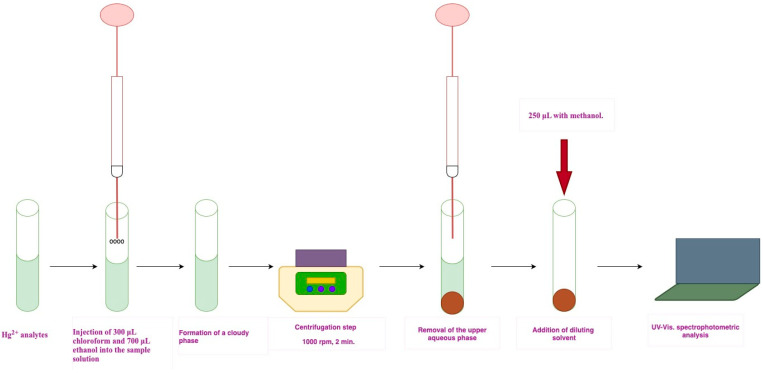
Graphical representation of the proposed DLLME procedure.

250 μL of Fe(II)Pc (5.00 × 10^−6^ M, methanol) was added as the sensor to 5 mL of an aqueous sample solution containing Hg^2+^ in a 10 mL conical glass tube. The pH was then adjusted to 2.00 using a citric acid–sodium hydroxide buffer. An extraction mixture consisting of 300 μL chloroform (as the extraction solvent) and 700 μL ethanol (as the dispersive solvent) was then rapidly injected into the sample solution with the aid of a 5 mL syringe. A fine cloudy solution was formed and subsequently centrifuged at 1000 rpm for 2 min. Following centrifugation, the aqueous phase was completely discarded, and the sedimented analyte-rich phase was diluted to 250 μL with methanol. Finally, absorbance measurements were carried out at 276 nm using a quartz cell (optical path length: 1 cm, volume: 250 μL) (Figure 7).

## 4. Conclusions

Mercury and its compounds, despite their well-documented toxicity, remain extensively employed in industrial applications, thereby necessitating reliable methods for their determination in environmental samples. Conventional DLLME based approaches often depend on advanced techniques such as HPLC, GC, ICP, and AAS, which are costly and require specialized expertise. In this study, a novel DLLME method integrated with spectrophotometry was developed for the determination of Hg^2+^ in water samples using Fe(II) phthalocyanine as a sensor. Phthalocyanine compounds are well recognized as excellent candidates for sensor design due to their structural stability and strong coordination ability [61]. A review of the literature indicates that reports utilizing phthalocyanine compounds as sensing or complexing agents in DLLME are scarce. Therefore, the use of a phthalocyanine derivative with a high capacity to form stable and selective sensor systems significantly enhances the novelty and originality of the present work. Furthermore, the proposed method aligns with the principles of green chemistry by minimizing organic solvent consumption and waste generation. Although it exhibits a relatively narrow linear range compared to instrumental techniques, it offers low LOD and LOQ values and a high enrichment factor. In addition, its implementation through a simple and accessible technique such as UV–Vis spectrophotometry makes it suitable for routine analytical applications.

The concentration of the Fe(II)Pc ligand to be used in the DLLME method for the spectrophotometric determination of Hg^2+^ was established as 5.00 × 10^−6^ M, and 276 nm, where the highest absorbance was observed, was selected as the working wavelength. The proposed method is extremely simple and rapid to apply. The rapid stabilization of the formed complex and the short centrifugation time are important factors contributing to their applicability.

The formation between Hg^2+^ and Fe(II)Pc complex was confirmed by a noticeable red shift in the UV–Vis spectrum, where the main Q-band moved from 718 nm to 728 nm upon the addition of Hg^2+^ ions (Supplementary Figure S3). This spectral response clearly indicates coordination between mercury ions and the nitrogen atoms of the phthalocyanine macrocycle [73]. The interaction causes electronic perturbation within the conjugated π-system, consistent with literature reports describing the affinity of soft metal ions such as Hg^2+^ for nitrogen donor sites [74]. Furthermore, Job’s plot analysis supported a 1:1 metal-to-ligand stoichiometry (Supplementary Figure S4), validating the proposed binding mechanism between Fe(II)Pc and Hg^2+^ [75].

## Figures and Tables

**Table 1:** Tolerance limits of interfering for the determination of Hg^2+^ (6.32 µg/L).

Foreign Ion Added	Tolerance Limit (µg/L)
Ag^+^	132
Cu^2+^	119
Cr^3+^	158
Co^2+^	94
Pb^2+^	51
Ni^2+^	51
Cd^2+^	299
Mn^2+^	106
Zn^2+^	427
NO_3_^−^	556
ClO_4_^−^	435
SO_4_^2−^	214
PO_4_^3−^	278
H_2_PO_4_^−^	223

**Table 2:** Analytical performance parameters for Hg^2+^ determination.

Parameters	
Linear range, µg/L	1.00–20.00
Correlation coefficient (R^2^)	0.9991
LOD, µg/L	1.44
LOQ, µg/L	4.80
Enhancement factor,	105
RSD, % (*n* = 6)	1.27–2.68 ^a^ and 1.09–3.01 ^b^

^a^ Intra-day precision; ^b^ Inter-day precision.

**Table 3:** Accuracy evaluation of the DLLME method by recovery studies in water samples.

Sample	Hg^2+^ Amount µg/L (*n*:3)		
	Added	Finded ± SD	Recovery %
Black Sea	8.00	7.49 ± 0.11	94.00
Tap water	8.00	7.92 ± 0.04	99.00
Sera Lake	8.00	8.17 ± 0.13	102.12

Results are expressed as mean ± SD of three independent samples, each analyzed in triplicate.

**Table 4:** Comparative Assessment of the Developed DLLME Method and Other DLLME Strategies for Hg^2+^ Determination.

Extraction Method	Linear Range (µg/L)	LOD (µg/L)	Enrichment Factor	RSD (%)	Reference
DLLME	1.00–100	0.30	200	2.80–7.50	[5]
IL-DLLME	12.00–140	3.90	18.80	1.70	[70]
DLLME ^a^	10.00–200	3.00	-	10.00–12.00 ^a^	[57]
DLLME	0.50–100	0.15	39	2.60	[71]
IL-DLLME	0.050–1.00 ^b^	0.010 ^b^	128	0.78	[72]
DLLME	1.00–20	1.44	105	1.27–2.68 ^c^ and 1.09–3.01 ^d^	Present study

^a^ RSD (%) for the Hg^2+^; ^b^ The linear range was reported in µg/mL; ^c^ Intra-day precision; ^d^ Inter-day precision.

## Data Availability

The original contributions presented in this study are included in the article. Further inquiries can be directed to the corresponding author.

## Supplementary Materials

The following supporting information can be downloaded at: https://www.mdpi.com/article/10.3390/molecules30214192/s1.
