# Supplementary material for: A Novel DLLME-Based Approach for the Spectrophotometric Determination of Mercury in Environmental Samples Using the Fe(II) Phthalocyanine Sensor
Source: Molecules. 2025 Oct 27;30(21):4192. doi: 10.3390/molecules30214192 (PMC12608134; doi:10.3390/molecules30214192)
Supplement: Supplementary file 1 [file molecules-30-04192-s001.zip › molecules-3937859-supplementary.pdf]

SUPPLEMENTARY INFORMATION

A Novel DLLME-Based Approach for the Spectrophotometric Determination of Mercury in Environmental Samples Using the Fe(II) Phthalocyanine Sensor

Yasemin Çağlar

Department of Genetic and Bioengineering, Giresun University, 28200 Giresun, Türkiye; yasemin.caglar@giresun.edu.tr; Tel.: +90-454-310-40-16

1. Equipment

All absorbance measurements were carried out on a Thermo Scientific Evaluation Array spectrophotometer (Thermo Fisher Scientific, Waltham, MA, USA) equipped with a quartz microcuvette (250 µL capacity), with instrument control provided via an Asus computer. Phase separation from aqueous solutions was facilitated using a Benchtop Centrifuges K2015R model centrifuge. The pH values were determined with a Hanna HI 2211 pH meter (Hanna Instruments, Woonsocket, RI, USA) fitted with a glass electrode. Dissolution of the Fe(II)Pc compound was assisted by employing a Kudos SK2210 HP ultrasonic bath (Kudos Instruments Corp., New York, NY, USA).

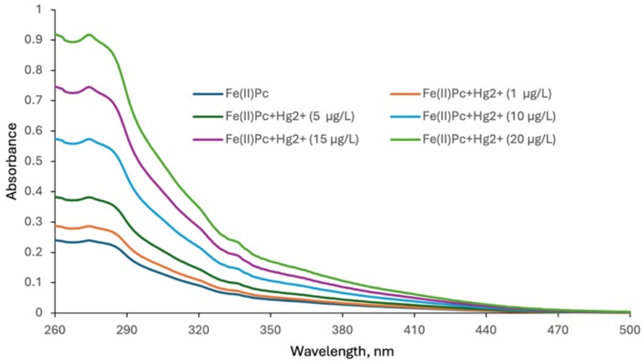

Supplementary Figure S1. Effects of Hg^{2+} ions on the absorbance of the Fe(II)Pc sensor in methanol. Ligand concentration = 5.00 × 10^{-6} M. Hg^{2+} concentration: 1.00–20 µg/L.

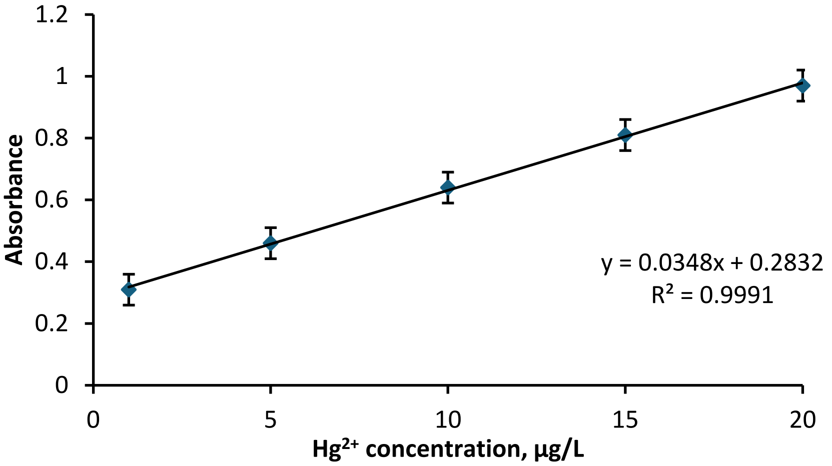

Supplementary Figure S2. The linear calibration curve of Hg^{2+} using the Fe(II)Pc based DLLME–Uv–Vis method.

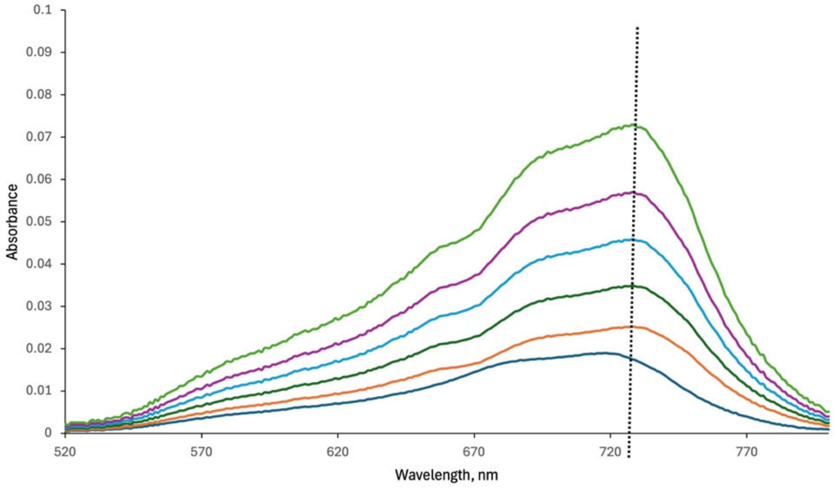

Supplementary Figure S3. Effect of Hg^{2+} ions on the Q-band absorption of the Fe(II)Pc sensor in methanol. Ligand concentration = 5.00×10^{-6} M; Hg^{2+} concentration: 1.00–20 μg/L.

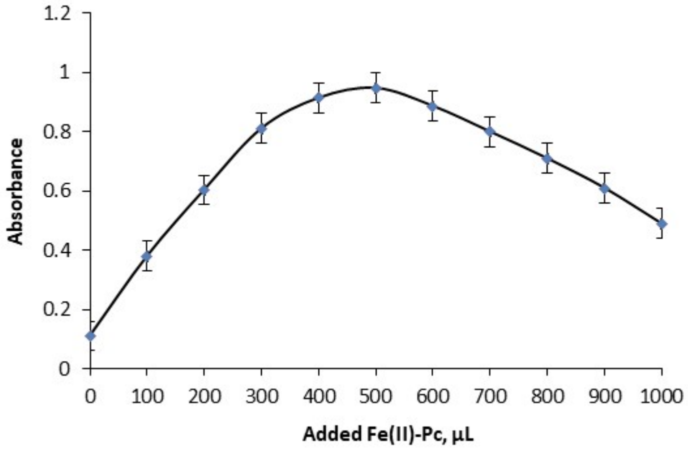

Supplementary Figure S4. Job's plot for determining the stoichiometry of the Hg^{2+}:Fe(II)-Pc complex.
